# Signal Denoising of Traffic Speed Deflectometer Measurement Based on Partial Swarm Optimization–Variational Mode Decomposition Method

**DOI:** 10.3390/s24123708

**Published:** 2024-06-07

**Authors:** Chaoyang Wu, Yiyuan Duan, Hao Wang

**Affiliations:** 1 School of Civil Engineering, Lanzhou Jiaotong University, Lanzhou 730070, China; wuchaoyang@lzjtu.edu.cn (C.W.); 12231289@stu.lzjtu.edu.cn (Y.D.); 2 Department of Civil and Environmental Engineering, Rutgers, The State University of New Jersey, Piscataway, NJ 08854, USA

**Keywords:** deflection test, VMD, PSO, TSD, denoising

## Abstract

To accurately identify the deflection data collected by a traffic speed deflectometer (TSD) and eliminate the noise in the measured signals, a TSD signal denoising method based on the partial swarm optimization–variational mode decomposition (PSO–VMD) method is proposed. Initially, the VMD algorithm is used for modal decomposition, calculating the correlation coefficients between each decomposed mode and the original signal for modal selection and signal reconstruction; Then, the particle swarm optimization algorithm is utilized to optimize the number of modes K and the value α for the VMD algorithm, adopting fuzzy entropy as the affinity function to circumvent effects from sequence decomposition and forecasting accuracy, thus identifying the optimal combination of hyperparameters. Finally, the analysis on simulated signals indicates that the PSO–VMD method secures the best parameters, showing a clear advantage in denoising. Denoising real TSD data validates that the approach proposed herein achieves commendable outcomes in TSD deflection noise reduction, offering a feasible strategy for TSD signal denoising.

## 1. Introduction

Assessing the structural integrity of pavement systems is crucial for ensuring safe and reliable transportation infrastructure. To achieve this, nondestructive testing tools equipped with multiple sensors are increasingly utilized. Among these tools, the falling weight deflectometer (FWD) stands out as a valuable device for evaluating the structural condition of pavement layers [1]. Additionally, the traffic speed deflectometer (TSD) serves as another nondestructive pavement evaluation tool, proficient in efficiently and effectively assessing the structural integrity and load-bearing capacity of roadways under normal traffic flow [2]. Equipped with multiple Doppler sensors, the TSD monitors pavement deflection and deformation in real time while traveling at highway speeds. Its reliability has been validated through applications such as the SHRP2 project [3]. However, during data collection, sensors often encounter noise issues due to external environmental influences and testing complexity, leading to deviations between test results and true signal characteristics. To ensure enhanced precision, data collected by Doppler sensors undergo preprocessing, employing effective techniques to extract genuine signals from ambient noise, thereby ensuring the precision and dependability of the data analysis [4].

The issue of TSD sensor signal noise has garnered significant attention, prompting the adoption of traditional methods for signal denoising. In the UK, traffic speed deflectometer (TSD) measurements are often averaged to a predetermined length as a denoising strategy, albeit subjectively, potentially resulting in the loss of meaningful information [5]. Similarly, the rolling wheel deflectometer employs moving average techniques to mitigate rolling noise [6]. Katicha et al. introduced a smoothing spline regression method to estimate true deflection slope in TSD deflection slope measurements [7]. Katicha et al. [4] proposed using the difference order method to evaluate changes in pavement structure and spatial variability caused by Doppler sensors’ noise, and provided a method to assess the standard deviation of sensor noise. They also used the unbiased risk estimator method to determine the optimal smoothness of TSD deflection slopes, and compared this method with the generalized cross-validation method [8], with results showing the unbiased risk method to be superior to the generalized cross-validation method. However, TSD actual measurement data present non-stationary signals, with signal frequencies evolving over time, rendering conventional methods ineffective. Initial attempts at filtering TSD deflection slope data utilized wavelet filtering, revealing instability in calculating the structural number directly from TSD test data [9]. Katicha et al. suggested the need for wavelet transform methods to denoise the noise in the TSD’s collected data. After comparing and analyzing hard threshold, soft threshold, and classical Bayesian thresholding methods, it was considered that the classical Bayesian method is more suitable for project-level deflection testing denoising, while hard and soft thresholding methods are suitable for network-level deflection testing noise reduction applications. However, given the variety in wavelet basis functions, thresholding methods, and undefined decomposition scales, the algorithm itself lacks adaptive characteristics, leading to a certain degree of uncertainty in its application.

The introduction of advanced signal decomposition methods has significantly enhanced the analysis and processing of complex signals. Huang [10] innovatively introduced the concept of intrinsic mode functions (IMFs) in 1996, involving the analysis method of empirical mode decomposition (EMD) that decomposes any signal into a series of intrinsic modalities. The EMD method is a significant improvement over traditional time-frequency analysis methods that require the predetermination of base functions. As an adaptive time-frequency localized analysis method, it allows for the decomposition of signals into multiple intrinsic mode functions based on their own time scale characteristics, without the need for pre-setting orthogonal base functions. Nonetheless, EMD faces issues with modal mixing during decomposition, attributed to how signal extremities impact IMFs, leading to modal mixing in instances of uneven distribution. Flandrin and colleagues [11] performed statistical analyses on white noise decomposition via EMD and introduced an enhanced version of EMD based on noise-assisted analysis, termed the ensemble EMD (EEMD) method. EEMD somewhat ameliorates the issue of modal mixing, yet it demands extensive computational resources, and the shortcomings of EMD remain inadequately resolved.

In the search for more robust and mathematically grounded signal decomposition methods, variational mode decomposition (VMD) has emerged as a notable advancement [12]. VMD distinguishes itself from other modal analysis techniques through a solid mathematical grounding. It has rapidly found applications across diverse sectors [13,14,15,16,17] since its introduction. To date, VMD has seen successful application in fields like structural health monitoring [18], ground-penetrating radar signal analysis [19,20], and seismic signal processing [21]. Currently, the introduction of the VMD method for denoising TSD test data has not yet been reported. Additionally, the application of the VMD technique requires the optimization of just two initial parameters [22]. In this segment, the particle swarm optimization (PSO) strategy is employed to fine-tune these two preliminary parameters of the VMD method [23].

This study aims to fill this gap by employing the PSO algorithm for the optimization of VMD parameters, utilizing fuzzy entropy as the affinity measure. The denoising of TSD deflection speed signals is achieved through simulation and empirical validation. The purpose of this work is to develop an effective denoising method for TSD signals by optimizing VMD parameters using PSO and fuzzy entropy. This approach aims to improve the accuracy and reliability of TSD measurements, which are critical for evaluating road conditions and ensuring transportation safety. By addressing the challenges associated with parameter selection in VMD and demonstrating the method’s superiority over traditional techniques, this research contributes to the advancement of signal processing in the field of traffic monitoring.

## 2. Methods

### 2.1. TSD Noise Filtering Model

TSD actual measurements include inevitable sensor testing noise, with measurements deviating from true values. Observing from the distribution, outliers exhibit certain randomness, whereas noise distribution shows a regularity. In terms of numerical magnitude, outliers are significantly larger than adjacent data points, while noisy data differ slightly from the true value. Hence, data containing noise can be treated with filtering techniques during the data processing stage [5].

The working frequency of the TSD Doppler sensor is 1000 Hz. Assuming that the vehicle speed during TSD data collection is 80 km/h, the TSD sensor can capture signals at 22 mm intervals on the road surface, with 45 data points distributed over a length of 1 m. According to the central limit theorem, the collected data noise should follow a normal distribution pattern. If ξ the standard deviation is σ, then the standard deviation is $\sqrt{2}\sigma$. Thus, TSD data align with the noise filtration model, enabling noise removal through this model.

As indicated in Equation (1), the TSD data filtering model comprises actual values and noise data [4,5].

(1) $$y_{ij}=s_{i}+e_{ij}$$

In the formula, $y_{ij}$ represents the measurement of the *i*-th sensor during the *j*-th measurement by TSD, $s_{i}$ represents the true value measured by the *i*-th sensor, and $e_{ij}$ indicates the noise from the *i*-th sensor in the *j*-th measurement.

Consequently, as indicated in Equation (2), the error $\xi_{i}$ for the *i*-th Doppler sensor is calculated as follows:

(2) $$y_{i1}-y_{i2}=e_{i1}-e_{i2}=\xi_{i}$$

### 2.2. Principle of Variational Mode Decomposition

VMD, as a recently introduced adaptive method for decomposing multi-component signals, can break down complex signals composed of multiple frequencies into several IMFs [12]. The VMD decomposition process mainly consists of constructing and solving a variational problem. This construction is carried out in three steps: (1) Calculating the analytic signal related to each mode through Hilbert transformation; (2) Adjusting the estimated central frequencies of each mode by introducing exponential terms, thereby shifting each mode’s spectrum to the baseband; (3) Calculating the norm of the aforementioned demodulated signals. Continuous optimization of this function can achieve decomposition results akin to those of EMD decomposition.

Assuming the input signal y is decomposed into *K* intrinsic mode functions, with *k* = 1, 2, …, *K*:

(3) $$u_{k}(t)=A_{k}(t)\cos\left(\phi_{k}(t)\right)$$

where $A_{k}(t)$ denotes the instantaneous amplitude of $u_{k}(t)$; $d\phi_{k}(t)/dt=\omega_{k}(t)$, $\omega_{k}(t)$ indicates the instantaneous frequency of $u_{k}(t)$.

Assuming that each $u_{k}(t)$ has a specific central frequency and limited bandwidth, the variational challenge involves finding *K* intrinsic mode functions $u_{k}(t)$ under two constraints: (1) The sum of all modes equals the input signal; (2) The cumulative estimated bandwidth of the intrinsic mode functions is minimized. The variational problem with constraints is presented as follows:

(4) $$a=1,\;\left\{ \begin{array}{l} \begin{array}{c} \min\\ \left\{u_{k}\right\},\left\{\omega_{k}\right\} \end{array}\left\{{\sum_{k}\left\|\partial_{t}\left[\left(\sigma (t)+\frac{j}{\pi t}\right)*u_{k}(t)\right]e^{-j\omega_{k}t}\right\|}_{2}^{2}\right\}\\ \;\;\;\;\;\;\;\;\;\;\;\;\;\;\;\;\;\;\;\;\;\;\;\;\;\;\;\;\;\;\;\;\;\;\;\;s.t.\sum_{k=1}^{K}u_{k}(t)=y(t) \end{array}\right.$$

where $\left\{\mu_{k}\}=\{\mu_{1},\cdots ,\mu_{k}\},\;\{\omega_{k}\}=\{\omega_{1},\cdots ,\omega_{k}\right\}$.

For acquiring the optimal solution of the stated variational problem, a quadratic penalty factor *α* and Lagrange multiplier *λ* (*t*) are introduced, transforming the issue into an unconstrained variational problem as depicted in Equation (5):

(5) $$\begin{array}{ll} L\left(\left\{\mu_{k}\right\},\left\{\omega_{k}\right\},\lambda \right)= & \alpha \sum_{k}{\left\|\partial_{t}\left[\left(\partial (t)+\frac{j}{\pi t}\right)*\mu_{k}(t)\right]e^{-j\omega_{k}t}\right\|}_{2}^{2}+\\ & {\left\|f(t)-\sum_{k}\mu_{k}(t)\right\|}_{2}^{2}+\langle \lambda (t),f(t)-\sum_{k}\mu_{k}(t)\rangle \end{array}$$

The stated variational problem is solved using the alternating direction method of multipliers, updating $\mu_{k}^{n+1}$, $w_{k}^{n+1}$, and $\lambda_{n+1}$, to locate the saddle point of Equation (5), with the formula $\mu_{k}^{n+1}$ as follows:

(6) $$\mu_{k}^{n+1}=\underset{\mu_{k}\in X}{\arg\min}\left\{\alpha {\left\|\partial_{t}\left[\left(\partial (t)+\frac{j}{\pi t}\right)*\mu_{k}(t)\right]e^{-j\omega_{k}t}\right\|}_{2}^{2}+{\left\|f(t)-\sum_{i}\mu_{i}(t)+\frac{\lambda (t)}{2}\right\|}_{2}^{2}\right\}$$

where $\omega_{k}$ equals $\omega_{k}^{n+1};$ and $\sum_{i}\mu_{i}(t)$ equals $\sum_{i\neq k}\mu_{i}{\left(t\right)}^{n+1}$.

The solution for the quadratic optimization issue:

(7) $$u_{i}^{n+1}(\omega )=\frac{f-\sum_{i\neq K}u_{i}(\omega )+\frac{\lambda (\omega )}{2}}{1+2\alpha {\left(\omega -\omega_{i}\right)}^{2}}$$

Transitioning the central frequency determination issue into the frequency domain:

(8) $$\omega_{k}^{n+1}=\underset{\omega_{k}}{\arg\min}\left\{\int_{0}^{\infty }{\left(\omega -\omega_{k}\right)}^{2}{\left|{\hat{\mu }}_{k}(\omega )\right|}^{2}d\omega \right\}$$

The approach for updating the central frequency is derived as follows:

(9) $$\omega_{i}^{n+1}=\frac{\int_{0}^{\infty }\omega {\left|u_{i}(\omega )\right|}^{2}d\omega }{\int_{0}^{\infty }{\left|u_{i}(\omega )\right|}^{2}d\omega }$$

Consolidating the aforementioned concepts, the process for solving the variational problem is succinctly outlined as follows:

(a) Initialize $\left\{{\hat{u}}_{k}^{1}\right\},\left\{\omega_{k}^{1}\right\},\left\{{\hat{\lambda }}^{1}\right\}$ and *n*;
(b) Update $u_{k}$ and $\omega_{k}$ according to the equation;
(c) Update λ;
  (10) $${\hat{\lambda }}^{n+1}(\omega )\leftarrow {\hat{\lambda }}^{n}(\omega )+\tau \left[\hat{f}(\omega )-\sum_{k}{\hat{u}}_{k}^{n+1}(\omega )\right]$$
(d) Continue steps (b) to (c) until meeting the iteration criteria $\sum_{i}{\left\|{\hat{\mu }}_{k}^{n+1}-{\hat{\mu }}_{i}\right\|}^{2}{\left\|{\hat{\mu }}_{k}^{n}\right\|}_{2}^{2}<e$.

### 2.3. Principle of Particle Swarm Optimization Algorithm

The VMD algorithm, in acquiring IMFs, departs from the iterative sifting and signal stripping process utilized by the EMD method. However, it is more sensitive in the selection of optimization problem parameters [22]. The penalty factor (α) and the number of decomposition layers (K) in the VMD method need to be preset, which suggests that the combination of parameters can significantly influence the outcome. The penalty factor (α) in VMD controls the trade-off between data fidelity and smoothness of the decomposed modes. A higher α ensures that the modes closely follow the original signal, reducing noise separation efficiency, while a lower α may capture more noise. An optimal α balances signal fitting and smoothness to effectively separate signal and noise. The number of decomposition layers (K) determines the number of IMFs the signal will be decomposed into. Too few layers (low K) may result in broad modes that mix signal components, while too many layers (high K) may over-decompose the signal, capturing noise as separate modes. The optimal K ensures the clear separation of frequency components.

Therefore, it is necessary to find an appropriate method for the global optimization of parameters. PSO is a global optimization algorithm for parameter optimization, currently widely used in various parameter optimization analyses [23]. Its fundamental idea for finding the optimal solution is to achieve it through collaboration among different individuals within a swarm and the sharing of information among them. The algorithm is described as follows:

Let $x_{i}=(x_{i1},x_{i2},\cdots ,x_{in})$ represent the position of the *i*-th particle in an n-dimensional space; $p_{i}=(p_{i1},p_{i2},\cdots ,p_{in})$ represent its best experienced position; $p_{g}=(p_{g1},p_{g2},\cdots ,p_{gn})$ represent the best position experienced by all particles in the swarm; $v_{i}=(v_{i1},v_{i2},\cdots ,v_{in})$ represent the velocity of the *i*-th particle in the swarm. The change in the *d*-th dimension (1 ≤ *d* ≤ *n*) is as follows:

(11) $$\begin{array}{l} v_{id}(t+1)=wv_{id}(t)+c_{1}r_{1}(p_{id}-x_{id}(t))+c_{2}r_{2}(p_{gd}-x_{gd}(t))\\ \begin{array}{c} x_{id}(t+1)=x_{id}(t)+v_{id}(t+1)\\ w=w_{\max}-\frac{\left(w_{\max}-w_{\min}\right)T_{\max}}{T} \end{array} \end{array}$$

In the formula, w is the inertia weight and $T_{max}$ is the maximum number of iterations. $C_{1}$ and $C_{2}$ are learning factors, representing the acceleration weights that guide each particle toward $p_{i}$ and $p_{g}$ positions, respectively. $v_{id}(t)$ is the particle’s previous velocity; $c_{1}r_{1}(p_{id}-x_{id}(t))$ represents the “cognitive” part, indicating the particle’s own reasoning; $c_{2}r_{2}(p_{gd}-x_{gd}(t))$ represents the “social” part, indicating information sharing and cooperation among particles. In each dimension, particles have a maximum velocity limit $V_{\max}$. If the velocity in a certain dimension exceeds $V_{\max}$, then the velocity in that dimension is limited to $V_{\max}(V_{\max}>0)$. In this document, the population size N is 50, and the values of C1 and C2 are both 1.5. w is 0.5.

### 2.4. Fitness Function

**Figure 1:**
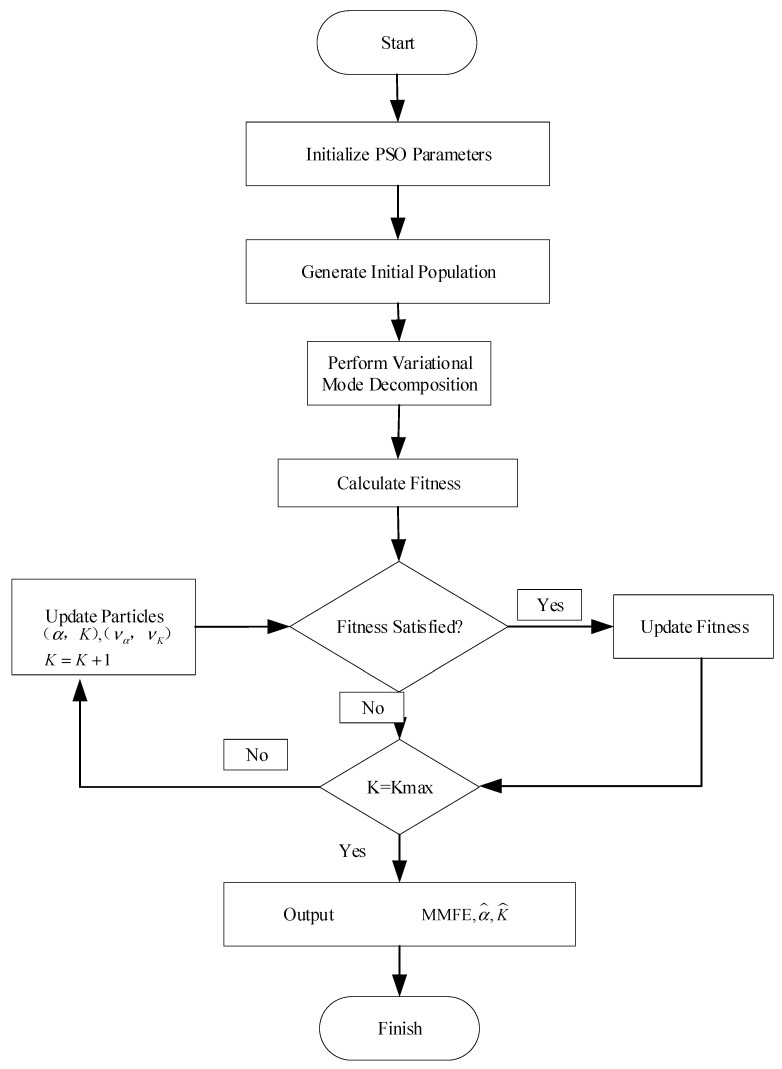
The flowchart of PSO–VMD method.

Figure 1 presents the flowchart of the proposed PSO–VMD method. The process begins with reading the TSD data after outlier removal, followed by denoising using the VMD method based on initialized PSO parameters. The PSO algorithm is employed to minimize the fitness function, which is defined based on the denoising performance of VMD. Fuzzy entropy is used as the affinity measure in the fitness function to evaluate the quality of the decomposed modes. The PSO algorithm starts with a population of potential solutions (particles), each representing a pair of K and α values. These particles are randomly initialized within predefined ranges. Each particle’s velocity and position are updated iteratively based on its own best position and the global best position found by the swarm. The velocity update considers both the cognitive component (individual particle experience) and the social component (swarm experience). The optimization process continues until convergence criteria are met. The optimal K and α values are obtained when the fitness function reaches its minimum value. Based on the aforementioned algorithmic concept, relevant programs were developed using Matlab 2018b [24].

It is understood that calculating the fitness function is a key step in the PSO–VMD method. Fuzzy entropy [25] (FuzzyEn) measures the probability of the emergence of new patterns; the larger the measure, the higher the probability of new patterns emerging, indicating a higher sequence complexity [26]. Therefore, multiscale fuzzy entropy is used as the fitness function in the PSO–VMD method. The specific process of the fitness function is as follows [27]:

(1) For a given N-dimensional time series [u(1),u(2),⋯,u(N)], time series undergo coarse-graining transformation, resulting in a new sequence:
  (12) $$x_{b}^{\left(\tau \right)}=\frac{1}{\tau }\sum_{c=(b-1)\tau +1}^{b\tau }x_{c},1\leq b\leq N/\tau$$

Here, *τ* represents a scaling factor. When *τ* = 1, $\left\{x_{b}^{\left(\tau \right)}\right\}$ corresponds to the original data. When *τ* > 1, the original data are divided into coarse-grained sequences $\left\{x_{b}^{\left(\tau \right)}\right\}$ with lengths not exceeding *N*/*τ*.

(2) Define the dimensionality m of the phase space (*m* ≤ *N* − 2) and the similarity tolerance r, and reconstruct the phase space:
  (13) $$X(i)=[u(i),u(i+1),\cdots ,u(i+m-1)]-u_{0}(i),i=1,2,\cdots ,N-m+1$$
  where $u_{0}(i)=\frac{1}{m}\sum_{j=0}^{m-1}u(i+j)$.
(3) Introduce a fuzzy membership function:
  (14) $$A(x)=\{\begin{array}{ll} 1, & x=0\\ \exp\left[-\ln(2){\left(\frac{x}{r}\right)}^{2}\right], & x>0 \end{array}$$
  where *r* is the similarity tolerance.

For i=1,2,⋯,N−m+1, calculate

$$A_{ij}^{m}=\exp\left[-\ln(2)\cdot {\left(d_{ij}^{m}/r\right)}^{2}\right],j=1,2,\cdots ,N-m+1,\;\mathrm{and}\;j\neq i$$

where the maximum absolute distance between window vectors (*X* (*i*) and *X* (*j*) is considered: $d_{ij}^{m}=d[X(i),X(j)]=\max_{p=1,2,\ldots ,m}\left(\left|u(i+p-1)-u_{0}(i)\right|-\left|u(j+p-1)-u_{0}(j)\right|\right)\;$.

(4) For each *i*, calculate its average to obtain:
  (15) $$C_{i}^{m}(r)=\frac{1}{N-m}\sum_{j=1,j\neq i}^{N-m+1}A_{ij}^{m}$$
(5) Define:
  (16) $$\Phi^{m}(r)=\frac{1}{N-m+1}\overset{N-m+1}{\underset{i=1}{\sum }}C_{i}^{m}(r)$$
(6) Therefore, the fuzzy entropy (*FuzzyEn*) of the original time series is as follows:
  (17) $$FuzzyEn(m,r)=\underset{N\to \infty }{\lim}\left[\ln\Phi^{m}(r)-\ln\Phi^{m+1}(r)\right]$$

For a finite dataset, the estimate of multiscale fuzzy entropy is as follows:

(18) $$MFE(m,\tau ,r,N)=FuzzyEn(m,\tau ,x_{a}^{\left(\tau \right)})$$

(19) $$FuzzyEn(m,r,N)=\ln\Phi^{m}(r)-\ln\Phi^{m+1}(r)$$

### 2.5. Evaluation Methods

To prove the effectiveness of the method, common denoising metrics such as root mean square error (*RMSE*), signal-to-noise ratio (*SNR*), and a smoothness index are compared with the wavelet transform method.

The *RMSE*, a common measure of the variance between the original and denoised signals, is defined as shown in Equation (20):

(20) $$RMSE=\sqrt{\frac{1}{N}\sum_{n=1}^{N}{\left[f(n)-\hat{f}(n)\right]}^{2}}$$

where f(n) represents the original signal and $\hat{f}(n)$ represents the denoised signal.

The *SNR* is traditionally used to assess the level of noise in test signals, with a higher *SNR* indicating better denoising performance:

(21) $$SNR=10\;\mathrm{lg}(p_{s}/p_{z})$$

where $p_{s}$ is the power of the original signal and $p_{z}$ is the power of the noise.

Sometimes, the *SNR* does not fully reflect the denoising effect. The smoothness index measures the smoothness of the signal after denoising, defined as follows:

(22) $$r=\frac{\left\{{\sum_{n+1}\left[\hat{f}(n+1)-\hat{f}(n)\right]}^{2}\right\}}{\left\{{\sum_{n+1}\left[f(n+1)-f(n)\right]}^{2}\right\}}$$

A higher *SNR* is indicative of a diminished presence of noise within the signal. Conversely, a lower *RMSE* suggests a greater fidelity of the denoised signal to its original, uncorrupted counterpart. Moreover, a reduced value of r denotes a more refined and smoother representation of the signal’s curve, enhancing its interpretability and utility in further analyses. These criteria help evaluate the effectiveness of denoising methods, providing a quantitative basis for comparing the proposed method with traditional wavelet transforms and other noise reduction techniques.

## 3. Numerical Experiments

When assessing the bearing capacity of pavement structures, the deflection information collected by the TSD is shown to be non-linear and non-stationary, making the extraction of effective signals a basis for further work.

To verify the noise reduction effect of the VMD method, a composite signal constructed here as shown in Equation (23) includes three sub-signals and a signal with Gaussian white noise, with a signal interval of 0.001 s and a total of 1000 points.

(23) f=cos(2π×5t)+1/4×(cos(2π×90t))+1/16(cos(2π×180t)+0.1×η

In the formula (23), $v_{1}=\cos(2\pi \times 5t)$ represents the first sub-signal;

$v_{2}=1/4\times (\cos(2\pi \times 90t))$ represents the second sub-signal;

$v_{3}=1/16(\cos(2\pi \times 180t)$ represents the third sub-signal;

η represents Gaussian white noise with a variance of 1.

**Figure 2:**
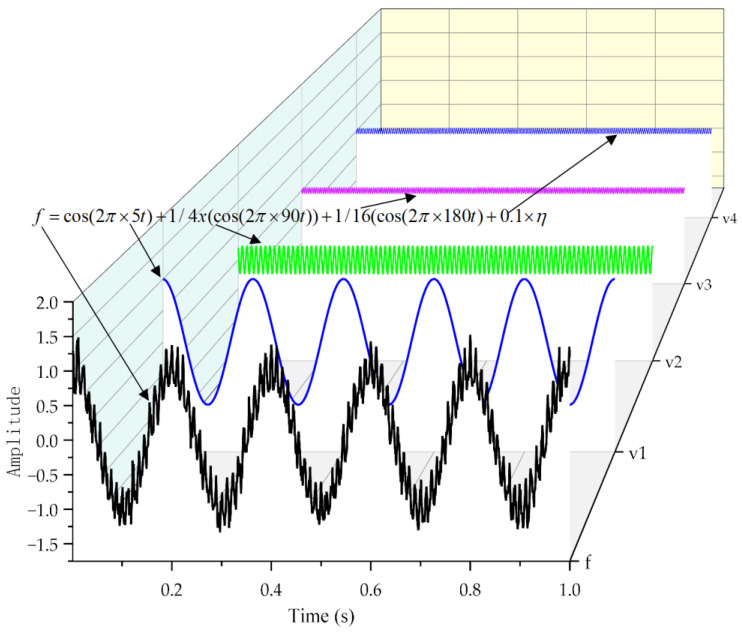
Synthetic signal and its four component signals.

As shown in Figure 2, the black signal is the composite signal f formed by the superposition of v1, v2, v3, and Gaussian white noise with a variance of 1. The blue signal represents the sub-signal v1, the green signal represents the sub-signal v2, and the purple signal represents the sub-signal v3. The signal amplitudes of v1, v2, and v3 are 1, 1/4, and 1/16, respectively.

### 3.1. Denoising Based on the VMD Method

By filtering the signal with added Gaussian white noise and comparing it with other filtering methods, we demonstrate the advantages of the VMD method in denoising. The VMD method is used to reduce noise. For VMD decomposition, the number of decomposition layers is chosen as three, with a commonly used penalty factor of 2000.

**Figure 3:**
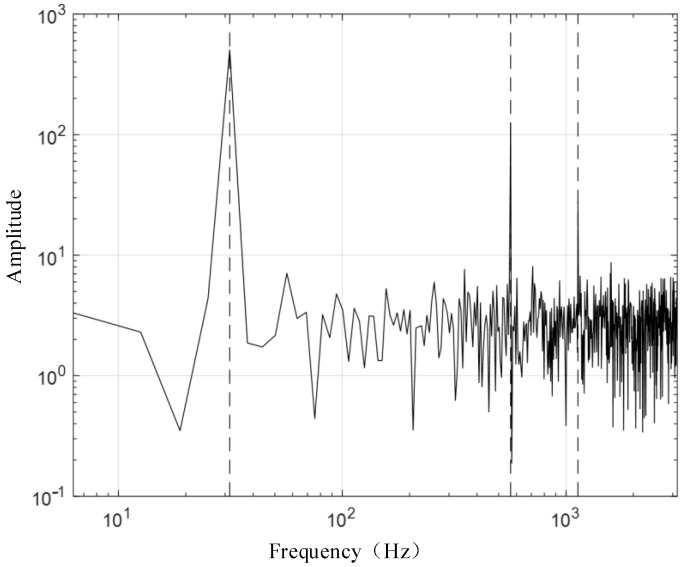
Input signal spectrum ($\left|{\hat{f}}_{n}\right|(\omega )$).

**Figure 4:**
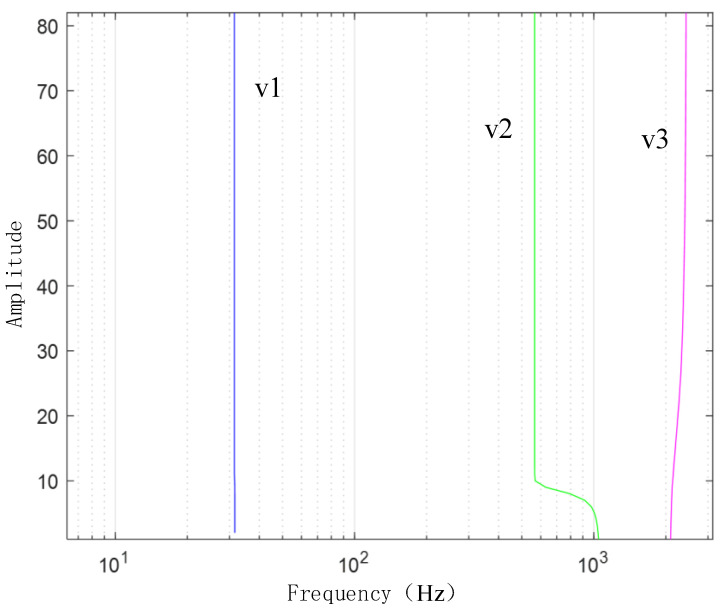
Evolution chart of the central frequencies ω.

**Figure 5:**
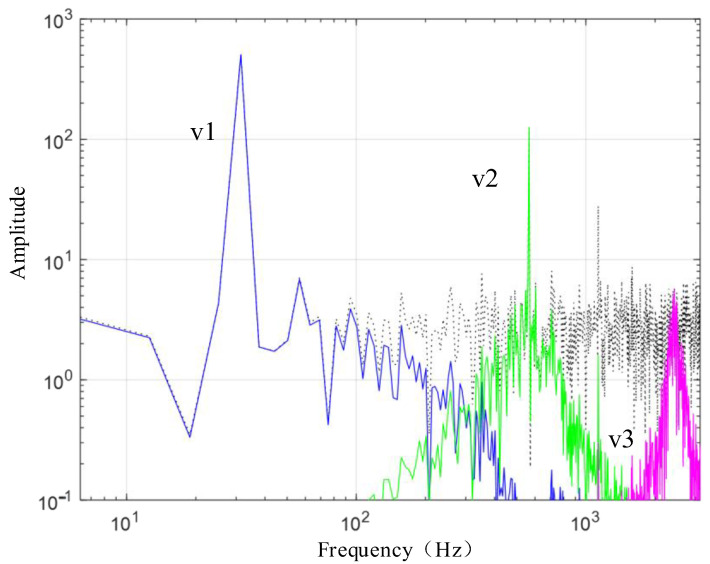
Spectral decomposition ($\left|{\hat{\mu }}_{k}\right|(\omega )$).

**Figure 6:**
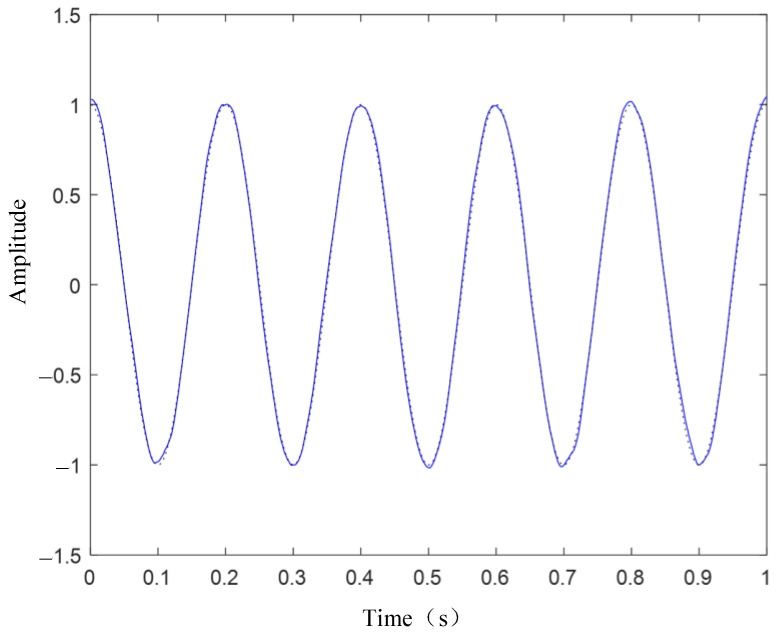
Original signal and recovered signal chart (v1).

**Figure 7:**
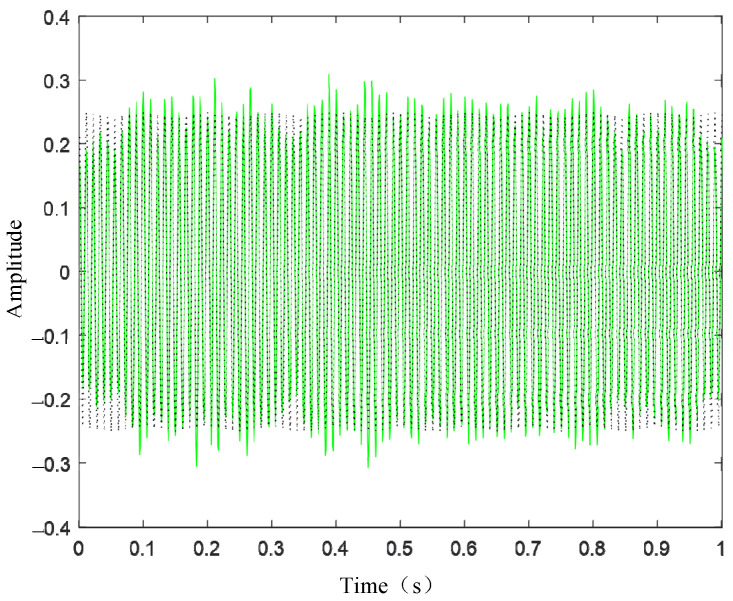
Original signal and recovered signal chart (v2).

**Figure 8:**
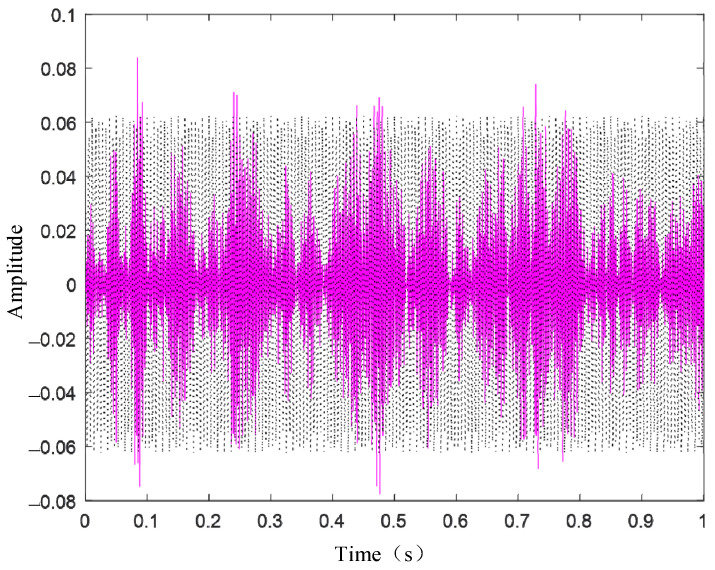
Original signal and recovered signal chart (v3).

Figure 3 presents the waveform spectrum of the original signal; Figure 4 shows the evolution of the central frequency; Figure 5 provides the spectral decomposition of the signal; Figure 6, Figure 7, and Figure 8, respectively, present comparison charts of the first, second, and third sub-signals of the original signal with the decomposed signal. The line colors in Figure 3, Figure 4, Figure 5, Figure 6, Figure 7 and Figure 8 have the same meanings as those in Figure 2.

The figures demonstrate that the VMD method can break down several components of a complex signal, with the first and second decomposed sub-signals closely matching the original signal. Although the third signal has a lower amplitude compared to the noise signal, the sub-signal with the smaller amplitude is also effectively reconstructed. Consequently, the VMD method can effectively decompose complex signals with multiple sub-signals, showing distinct advantages in its application to TSD measured signals.

### 3.2. Result

We utilize the VMD methods for original signal denoising, and then compare the root mean square error, signal-to-noise ratio, and smoothness index of the denoised and theoretical signals. Table 1 presents an academic and detailed translation focusing on the noise reduction capabilities of the VMD method, and the effectiveness and reliability of VMD in denoising complex signals, showcasing its advantages in practical applications.

Compared to the unoptimized VMD model, the PSO–VMD model showed a 3.07 dB increase in SNR, a 30.2% decrease in RMSE, and a 29.2% increase in the smoothness index.

## 4. Field Test Data Application Example

**Figure 9:**
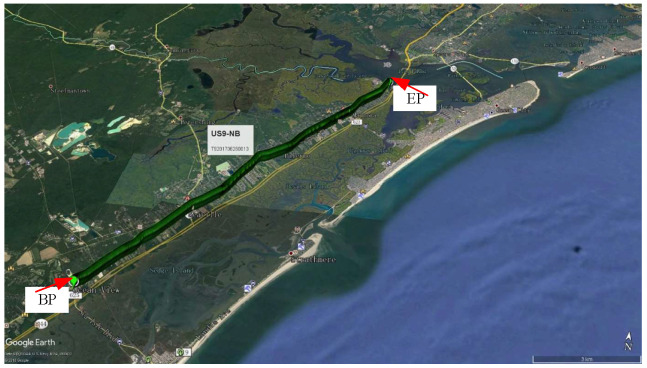
US9 within New Jersey. Google Earth™.

Taking the New Jersey section of US Route 9 (US9) as an example, we conduct TSD test signal preprocessing. As shown in Figure 9, the selected section is about 16,000 m long, with a data interval of 10 m, and about 1600 data points (starting from 0 m, ending at about 16,000 m).

### 4.1. Deflection Speed

**Figure 10:**
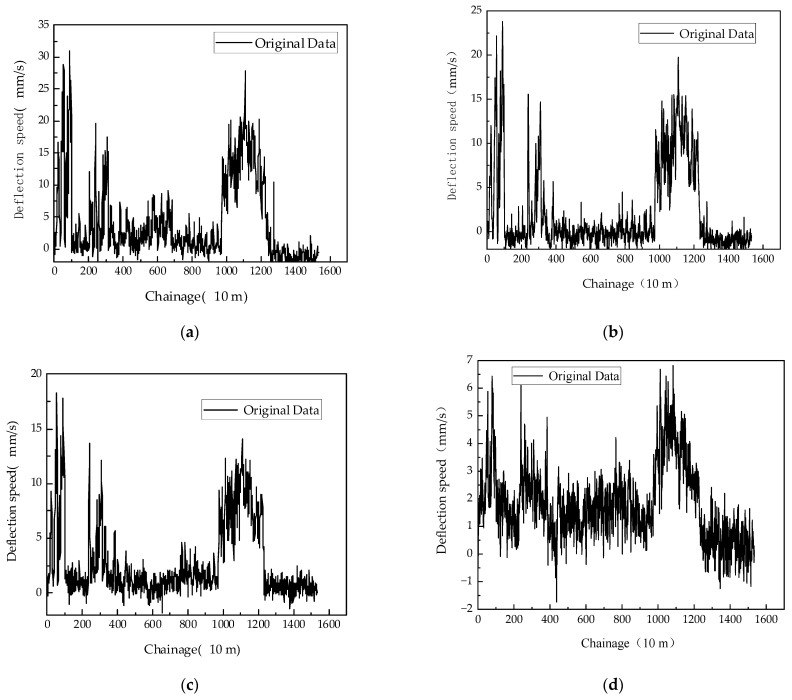

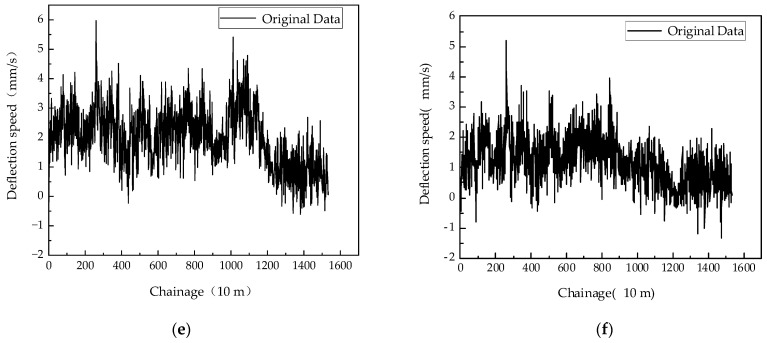
Original deflection speed of US9: (**a**) The readings from Doppler sensor S100; (**b**) The readings from Doppler sensor S200; (**c**) The readings from Doppler sensor S300; (**d**) The readings from Doppler sensor S600; (**e**) The readings from Doppler sensor S900; (**f**) The readings from Doppler sensor S1500.

The readings from Doppler sensors S100, S200, S300, S600, S900, and S1500 are shown in Figure 10.

### 4.2. Noise Reduction

Numerous natural phenomena generally conform to a normal distribution pattern. Extensive statistical evidence indicates that the deflection indices of the same type of pavement structure follow a normal distribution both at the initial completion stage and after a period of use. According to the central limit theorem, the overall sensor error conforms to a normal distribution. When analyzing TSD data, a 10 m interval is typically used, which is equivalent to sampling the population, so the errors still conform to a normal distribution [7]. For obtaining representative deflection values of the same pavement structure, values deviating from the normal range are excluded. Box plots have a certain advantage in identifying outliers. For outliers that do not conform to a normal distribution, the box plot method can be used for exclusion. Removing outliers before denoising is necessary to prevent them from unduly influencing the denoising process and potentially distorting the results.

**Figure 11:**
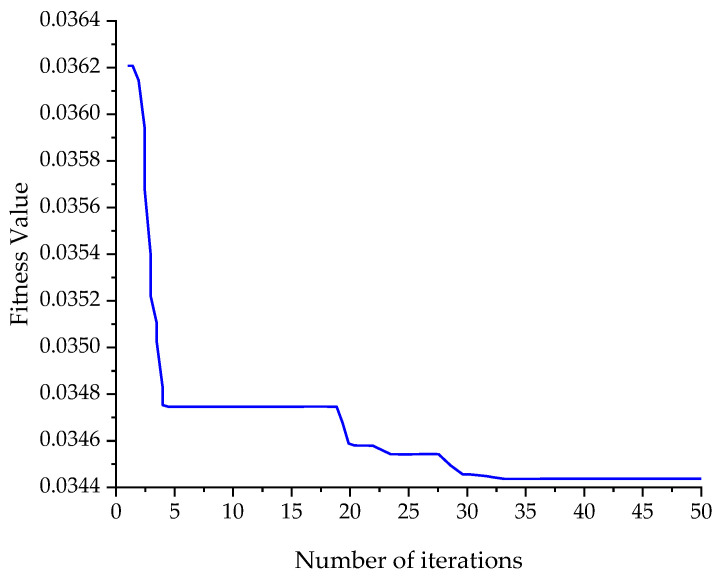
Fitness function curve.

After removing outliers from the TSD sensor’s actual measurement data, further data filtering is performed using the PSO–VMD method. Taking the S100 sensor as an example, after PSO optimization, a reasonable initial value for parameters is obtained as 4297, with the number of intrinsic mode functions being eight. The iterative process is shown in Figure 11, from which it can be observed that the value of the fitness function continuously decreases during the iteration process, trending toward stability.

**Figure 12:**
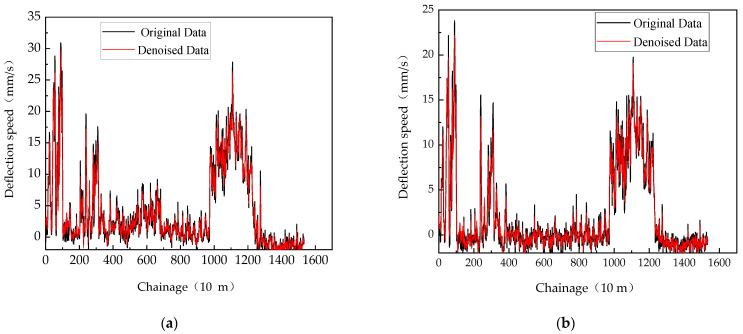

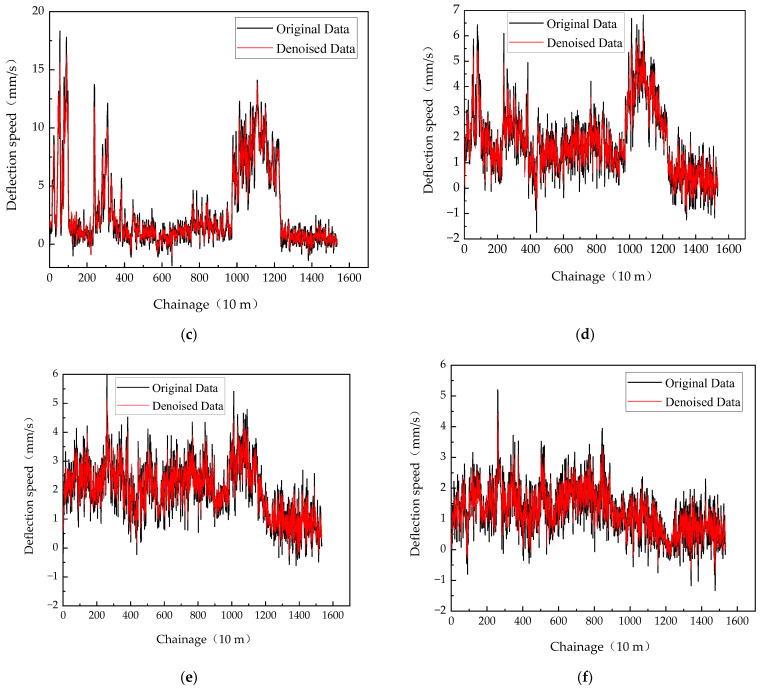
Deflection speed before and after denoising with PSO–VMD method. (**a**) The readings from Doppler sensor S100; (**b**) The readings from Doppler sensor S200; (**c**) The readings from Doppler sensor S300; (**d**) The readings from Doppler sensor S600; (**e**) The readings from Doppler sensor S900; (**f**) The readings from Doppler sensor S1500.

Due to the limited length of this article, the iteration processes for other sensor data follow similarly, and the iteration process of the fitness function is not listed here again. Following the noise elimination from the data of sensors S100, S200, S300, S600, S900, and S1500 on US Route 9, the curvature deflection speed is depicted in Figure 12.

The approach described in reference [9] is employed anew to compare the outcomes of denoising real signals, both pre and post-application of the PSO–VMD method. It can be seen that the proposed PSO–VMD method still surpasses the wavelet method in filtering effectiveness on actual data. As shown in Table 2, compared to the unoptimized wavelet method, the PSO–VMD model showed a 6.81 dB increase in SNR, a 54.3% decrease in RMSE, and a 85.0% increase in the smoothness index. It becomes evident that the proposed PSO–VMD method continues to outperform the wavelet method in terms of filtering efficiency on real-world data.

While this study primarily assumes Gaussian white noise, we recognize that engineering signals often contain a mixture of noise types, including non-Gaussian noise [28]. The method proposed in this paper, which uses particle swarm optimization (PSO) to optimize variational mode decomposition (VMD) parameters, aims to enhance the denoising process. However, the current scope of our research does not explicitly address the applicability of PSO–VMD to non-Gaussian noise types.

**Figure 13:**
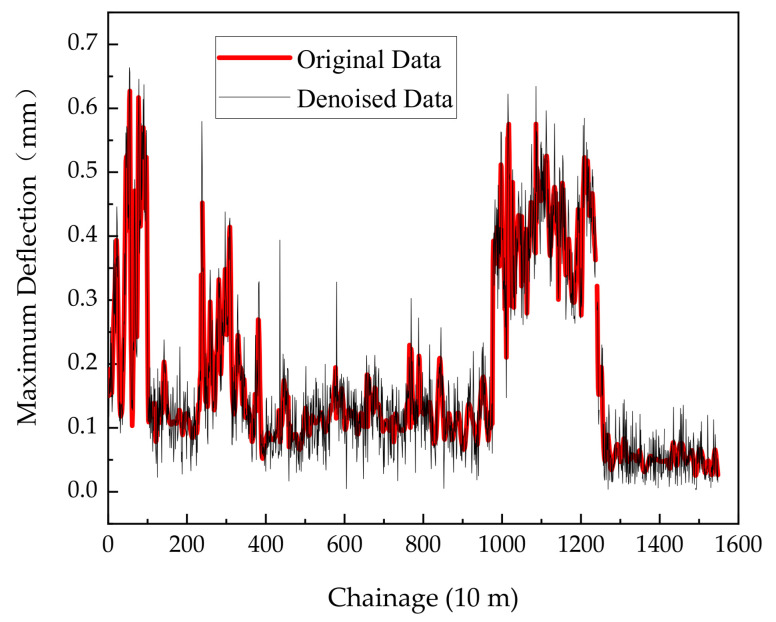
Maximum deflection curve after denoising.

The remaining data were fitted using the piecewise cubic hermite interpolating polynomial (PCHIP) method [29]. The result is a curve depicting the maximum deflection distributed along the mileposts of the US9 highway NB section, as shown in Figure 13.

## 5. Conclusions

This paper addresses the issue of manually setting parameters K and α in VMD by employing a PSO–VMD-based method, achieving noise reduction for both simulated signal data and in situ TSD Doppler sensor data.

(1) Utilizing variational mode decomposition (VMD), grounded in robust mathematical principles and offering rational outcomes, for denoising the original data post-error exclusion. The PSO optimization algorithm was employed to refine the hyperparameters K and α of variational mode decomposition, effectively addressing the challenge of manually determining K and α in conventional variational mode decomposition techniques.
(2) Conducting numerical experiments and comparing real data measurements with the wavelet transform method, the PSO–VMD method is adaptive and can significantly improve the signal-to-noise ratio and root mean square error of the data, resulting in better noise reduction. The PSO–VMD method mentioned in this paper can effectively eliminate noise from TSD Doppler sensors.

Future research can focus on evaluating and improving the PSO–VMD method’s performance in the presence of various noise types. This includes conducting extensive experiments with synthetic and real-world signals containing both Gaussian and non-Gaussian noise components [30]. Additionally, deflection speed analysis considering variations across the width of the road is also suggested. This will help in providing a more comprehensive understanding of pavement behavior and enhance the applicability of the PSO–VMD method in real-world scenarios.

## Figures and Tables

**Table 1:** Comparison of denoising effects using different methods on a synthetic signal.

Indicator	Original	VMD	PSO–VMD
r	0.3823	0.4225	0.5459
RMSE	0.1020	0.0787	0.0549
SNR	17.1779	19.4284	22.4988

**Table 2:** Comparison of denoising effects using different methods on a field signal.

Indicator	Wavelet (sym2) [9]	PSO–VMD
r	0.0604	0.4019
RMSE	0.1777	0.0811
SNR	12.3556	19.1676

## Data Availability

No new data were created.
